# Complementing reversed-phase selectivity with porous graphitized carbon to increase the metabolome coverage in an on-line two-dimensional LC-MS setup for metabolomics

**DOI:** 10.1039/c5an00206k

**Published:** 2015-05-21

**Authors:** Karin Ortmayr, Stephan Hann, Gunda Koellensperger

**Affiliations:** a Department of Chemistry , University of Natural Resources and Life Sciences (BOKU) Vienna , Muthgasse 18 , 1190 Vienna , Austria; b Institute of Analytical Chemistry , University of Vienna , Faculty of Chemistry , Waehringer Str. 38 , 1090 Vienna , Austria . Email: gunda.koellensperger@univie.ac.at ; Fax: +43 1 42779523

## Abstract

Efficient and robust separation methods are indispensable in modern LC-MS based metabolomics, where high-resolution mass spectrometers are challenged by isomeric and isobaric metabolites. The optimization of chromatographic separation hence remains an invaluable tool in the comprehensive analysis of the chemically diverse intracellular metabolome. While it is widely accepted that a single method with comprehensive metabolome coverage does not exist, the potential of combining different chromatographic selectivities in two-dimensional liquid chromatography is underestimated in the field. Here, we introduce a novel separation system combining reversed-phase and porous graphitized carbon liquid chromatography in a heart-cut on-line two-dimensional setup for mass spectrometry. The proposed experimental setup can be readily implemented using standard HPLC equipment with only one additional HPLC pump and a two-position six-port valve. The method proved to be robust with excellent retention time stability (average 0.4%) even in the presence of biological matrix. Testing the presented approach on a test mixture of 82 relevant intracellular metabolites, the number of metabolites that are retained could be doubled as compared to reversed-phase liquid chromatography alone. The presented work further demonstrates how the distinct selectivity of porous graphitized carbon complements reversed-phase liquid chromatography and extends the metabolome coverage of conventional LC-MS based methods in metabolomics to biologically important, but analytically challenging compound groups such as sugar phosphates. Both metabolic profiling and metabolic fingerprinting benefit from this method's increased separation capabilities that enhance sample throughput and the biological information content of LC-MS data. An inter-platform comparison with GC- and LC-tandem MS analyses confirmed the validity of the presented two-dimensional approach in the analysis of yeast cell extracts from *P. pastoris*.

## Abstract 2

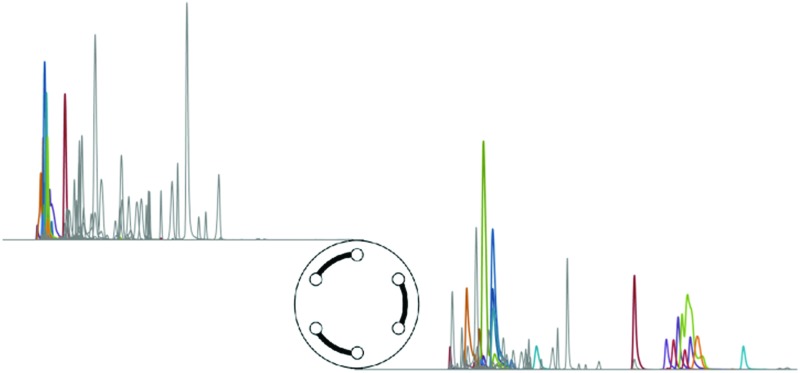
A novel on-line combination of reversed phase and porous graphitized carbon liquid chromatography increases the versatility in non-targeted metabolomics.

## Introduction

Liquid chromatography-mass spectrometry (LC-MS) and gas chromatography-mass spectrometry (GC-MS) are still the most widely employed analytical platforms in metabolomics.^1–6^ Despite the rapid evolution of high-resolution mass spectrometry instrumentation and the development of very high throughput methodologies^7,8^ in the past decade, chromatographic separation is still a prerequisite for reliable metabolite analysis.^3^ In this context, intracellular metabolites are generally considered a challenging set of analytes, as they are subject to rapid turnover and have widely different physicochemical properties and abundances within the cell.^6,9–11^ Moreover, typical cell extract samples are of high complexity and give rise to extensive ion suppression and matrix effects. As a consequence, the analytical method itself must tolerate samples with a dominant matrix whilst providing robustness and delivering the analytes to the mass spectrometer in a suitable solvent.

To date, a single analytical technique with comprehensive coverage of the intracellular metabolome does not exist.^11–13^ However, several attempts have been made on the establishment of such analytical platforms. For instance, van der Werf reported on the combination of six different complementary GC-MS and LC-MS methods that enabled the analysis of 380 compounds relevant in microbial metabolomics.^14^ While such a multi-method approach is presumably the only feasible way to achieve comprehensive coverage across the multiple compound classes in the intracellular metabolome, the collective evaluation of the resulting different data sets remains difficult.^13,15^ This is an aspect of high importance in non-targeted metabolomics, where the aim is the creation of metabolic fingerprints, *i.e.* representative snapshots of the metabolome in a certain condition. Moreover, each sample has to be analyzed with each method, which has a multiplicative effect both on the total analysis time and the data volume.

All of the aforementioned aspects have propelled efforts towards two-dimensional (2D) chromatography, where orthogonal separation methods are combined to give the maximum peak capacity and resolution. Although the concept of two-dimensional chromatography was already introduced decades ago,^16–19^ its establishment as standard tool in analytical laboratories was hampered by it being more demanding in terms of instrumentation, method development and data analysis. The potential benefits of such a methodology for the field of metabolomics span from reduced total analysis time to a significant simplification of data processing, all owing to the fact that the information is provided by a single analytical platform. Moreover, higher resolving power and coverage across different classes of metabolites can be achieved. Reversed-phase (RP),^20–22^ ion pair (IP)^14,23–26^ and hydrophilic interaction liquid chromatography (HILIC)^14,25,27–30^ are the most commonly applied chromatographic modes in the field of MS-based metabolomics. Moreover, methods employing silica hydride-based,^31–33^ mixed-mode^34,35^ and porous graphitized carbon (PGC)^36–38^ stationary phases have been described in literature. Ion pair LC provides sufficient selectivity to solve a variety of separation problems relevant in metabolomics, yet its practical application suffers from shortcomings in terms of robustness and contamination of the LC-MS instrumentation that limits its use for other purposes.^11^ While RP and HILIC represent the two most orthogonal chromatographic modes among those relevant for LC-MS based metabolomics, their coupling faces several challenges associated with mobile phase incompatibility. In most cases, suitable interfaces make use of trapping devices or make-up flows to adjust the solvent composition, resulting in an effective dilution at the expense of sensitivity. Nevertheless, the combination of RP and HILIC is invaluable in metabolomics and efficient methodologies have been described.^39,40^ Ultimately, even such separation systems face limitations with respect to resolving power, as important compound groups (*e.g.* sugar phosphates) of high relevance in metabolomics remain unresolved. Despite the potential benefits of utilizing other combinations of available chromatographic modes to obtain a higher degree of metabolome coverage, 2DLC and selectivities beyond RP and HILIC are rarely employed in LC-MS based metabolomics.

In current practice of metabolomics, reversed-phase liquid chromatography is still the chromatographic mode of choice. In spite of the typically poor retention of polar and ionic species, its broad metabolome coverage and compatibility with aqueous samples as well as the robustness and availability of different chemistries, column and particle geometries is unsurpassed with respect to other chromatographic modes.^2^ Nevertheless, the analysis of typical cell extract samples in RPLC-MS neglects a substantial fraction of weakly retained analytes, among them metabolites of high biological relevance. Despite the advancements in high-resolution mass spectrometry, several groups of isomeric and isobaric metabolites hence remain unaddressed. Moreover, good chromatographic separation is of supreme importance for metabolite annotation and identification, where in-source fragmentation increases the risk for misidentification and inaccurate quantification.^41^ This work evaluates the potential of porous graphitized carbon as complementary chromatographic selectivity for the separation of isomeric and isobaric molecules, especially sugar phosphates and related metabolites. To this end, a novel heart-cutting separation system based on the on-line two-dimensional combination of RP and PGC is introduced, in which the low-retained fraction from RPLC is transferred and separated on PGC. The resulting method is particularly convenient to implement as it makes use of standard HPLC equipment and requires only one standard two-position six-port valve for modulation and an additional HPLC pump. The applicability and benefits of this approach for both targeted and non-targeted metabolomics studies is demonstrated, as it provides improved resolution for biologically important isobars like sugar phosphates.

## Experimental section

### Metabolite standards

Sedoheptulose 7-phosphate and erythrose 4-phosphate were purchased from Carbosynth, cysteinylglycine from Bachem AG, and l-aspartic acid, citric acid monohydrate, l-glutamic acid, l-glutamine, glycine, l-leucine, dl-malic acid, l-methionine and l-valine from Merck. All other standard substances were obtained from Sigma Aldrich. A mix of 82 relevant intracellular metabolites was prepared from standard substances. Single standard solutions of each metabolite were obtained by exact weighing and dissolving the standard substance in a suitable solvent (0.1 M hydrochloric acid, 0.1 M sodium hydroxide or water). Equimolar mixtures of the 82 metabolites were used for initial method development. For the use as calibration standards, appropriate dilutions of equimolar stock mixes of the 82 metabolites were spiked with a defined volume of uniformly ^13^C-labeled yeast cell extract (prepared in-house^42^).

### *Pichia pastoris* culture and metabolite extraction

*P. pastoris* wildtype was grown in 3 parallel batch cultures in 1000 mL shake flasks with glycerol as carbon source. The samples were drawn 19 h after inoculation, immediately quenched in the 5-fold volume of quenching solution (60 : 40 methanol–water, –27 °C) and aliquoted by vacuum filtration using cellulose acetate filters (0.45 μm, Sartorius Stedim). The filters were kept at –80 °C until extraction. Metabolite extraction was accomplished using boiling ethanol extraction, as described in detail elsewhere.^42^ A uniformly ^13^C-labeled cell extract was added to each filter immediately prior to extraction. The cell-free ethanolic extracts were aliquoted for RP-PGC-TOFMS, RPLC-MS/MS and GC-MS/MS, dried under reduced pressure using a GeneVac EZ-2 solvent evaporation system and stored at –80 °C until further use. The aliquots for LC-MS/MS and RP-PGC-TOFMS analysis were reconstituted in an appropriate volume of LC-MS grade H_2_O immediately prior to analysis.

### Reversed-phase liquid chromatography

A silica-based Atlantis T3 C18 column (2.1 × 150 mm, 3 μm particle size, Waters) was used for reversed-phase separation. The flow rate was set to 250 μL min^–1^ and the column temperature to 45 °C. The injection volume was 5 μL in all cases. All solvents used for mobile phase preparation were of LC-MS grade and obtained from Sigma Aldrich. Mobile phase A consisted of water with 0.1% formic acid and 1% acetonitrile, mobile phase B was prepared from acetonitrile with 0.1% formic acid and 1% water. Initial gradient conditions (100% A) were maintained for 2 min, followed by a gradient from 0% to 95% B in 13 min. Including a cleaning step at 95% B and column re-equilibration at initial gradient conditions, the total run time was 20 min.

### Liquid chromatography on porous graphitized carbon

A Hypercarb PGC column (2.1 × 150 mm, 5 μm particle size, Thermo Scientific) was employed for second-dimension separation. It was operated at a flow rate of 250 μL min^–1^ and a column oven temperature of 45 °C. The injection volume was 5 μL (when used without coupling to RP). All solvents used for mobile phase preparation were of LC-MS grade and obtained from Sigma Aldrich. Mobile phase A consisted of water with 1% acetonitrile. Mobile phase B contained 90% water and 10% formic acid. Initial gradient conditions (99% A, 1% B) were maintained for 2.5 min, followed by a gradient from 1% to 40% B in 11.5 min. Including a cleaning step at 40% B and column re-equilibration at initial gradient conditions, the total run time was 20 min.

### Setup for RP-PGC liquid chromatography

**Fig. 1:**
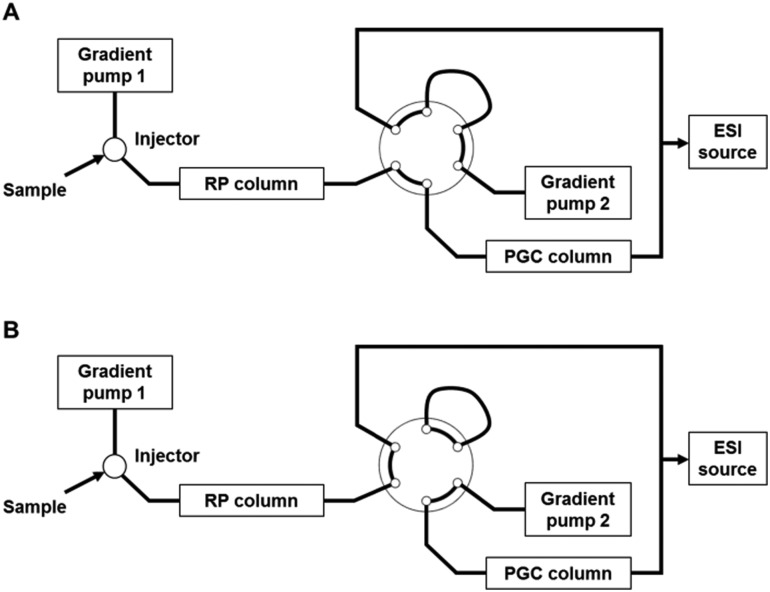
Setup for RP-PGC-ESI-MS. A two-position six-port valve is employed as switching valve to mediate the fraction transfer. The valve was set to position A for 0–2.5 min and position B between 2.5 and 20 min run time.

An Agilent 1290 Infinity HPLC system (first dimension) and an Agilent 1260 Bin Pump SL (second dimension) were used for coupling RP and PGC. The transfer of the initial fraction from the first-dimension column (RP) to the second dimension (PGC) was accomplished using a two-position six-port valve integrated as column switching valve in the thermostatically controlled column compartment. The sample was injected onto the RP column. From 0 to 2.5 min run time, the RP and PGC columns were serially coupled (position A, Fig. 1), so that all peaks eluting from the RP column during this time were automatically transferred and analyzed on the PGC column. The valve was switched to position B (Fig. 1) at 2.5 min, directing the second-dimension pump flow to the PGC column. RP and PGC separations were performed simultaneously by gradient elution. The column effluents are combined using a T-connector prior to introduction to the electrospray ion source.

### Mass spectrometry and data evaluation

An Agilent 6550 iFunnel Q-TOF mass spectrometer equipped with an Agilent Jet Stream electrospray ion source was used for the analysis of yeast cell extracts by RP-PGC-TOFMS. The source parameters were set as follows: 250 °C drying gas temperature, 13 L min^–1^ drying gas flow, 30 psig nebulizer pressure, 250 °C sheath gas temperature, 10 L min^–1^ sheath gas flow, 3500 V capillary voltage, 380 V fragmentor voltage. The TOF detector was operated in 2 GHz EDR mode with an acquisition rate of 3 spectra per second and 2695 transients per spectrum. Spectral data was recorded in the mass range of 60–1000 *m*/*z*. Data evaluation was performed using Agilent MassHunter Qualitative Analysis B.06.00 and MassHunter Quantitative Analysis B.07.00. Quantitation was based on peak areas obtained from extracted ion chromatograms (extraction width ± 20 ppm) within an external calibration strategy and internal standardization with fully ^13^C-labeled metabolite analogs. The calibration range was adjusted to the expected concentration of the respective metabolites in yeast cell extract samples, resulting in a working range of approximately 2 orders of magnitude for all metabolites and effective concentrations ranging from 0.4 μmol L^–1^ for low- to 2 mmol L^–1^ for high-abundant intracellular metabolites.

### Metabolite quantitation using RPLC-MS/MS and GC-MS/MS

LC-MS/MS and GC-MS/MS methods are routinely implemented in our laboratory for accurate quantitation of intracellular metabolites in cell extracts from various organisms. Quantitation is based on selected reaction monitoring (SRM) and the relative response to the respective fully ^13^C-labeled metabolites added to each sample *via* the U^13^C-labeled cell extract to account for errors introduced during sample-pretreatment, the analytical process as well as differences in ionization efficiency. Both methods have been validated, the corresponding analytical figures of merit are available elsewhere.^42,43^ For the purpose of this study, yeast cell extracts were analyzed on both platforms to provide reference values for comparison with RP-PGC-TOFMS.

RPLC-MS/MS was performed employing a silica-based Atlantis T3 C18 column (2.1 × 150 mm, 3 μm particle size, Waters) on a Thermo Accela 1250 HPLC system coupled to a Thermo TSQ Vantage triple quadrupole mass spectrometer. A detailed method description is available elsewhere.^42^

Quantification by GC-MS/MS was based on the on-time two-step derivatization (ethoxymation and trimethylsilylation) of intracellular metabolites using a Gerstel MPS2 dual rail Multi-Purpose Sampler followed by GC-MS/MS analysis using a Thermo TSQ Quantum XLS Ultra triple quadrupole GC-MS system. A detailed method description is available elsewhere.^43^

## Results and discussion

### Reversed-phase liquid chromatography in metabolomics

For metabolite separation, the best results were achieved employing a silica-based 100% wettable C18 stationary phase with water (0.1% formic acid) and acetonitrile (0.1% formic acid) as mobile phase compositions. Starting conditions employing a 100% aqueous mobile phase were a prerequisite for sufficient retention of moderately polar metabolites. Especially the separation of the critical isomer pairs citrate and isocitrate, 3′- and 5′-AMP as well as leucine and isoleucine is to be highlighted, as it is not readily accomplished even in ion pair LC. However, a substantial fraction of the sample elutes in or near the column void volume that is characterized by low separation efficiency and high matrix load leading to ion suppression in ESI-MS and therefore hindered identification and quantification of analytes. This fraction contains many polar and charged metabolites that are of high relevance for metabolomics, including pentose and hexose sugar phosphates as well as polar amino acids like arginine, asparagine, aspartic acid, cystathionine, glutamine, histidine, lysine and serine. Thus, in order to improve their reliable analysis, the employment of a different chromatographic selectivity was required.

### Liquid chromatography on porous graphitized carbon in metabolomics

All method optimization efforts focused on the separation of isobaric and isomeric sugar phosphates and related metabolites for which chromatographic separation is not readily achieved in MS-friendly conditions. As demonstrated previously,^44^ PGC shows good retention for sugar phosphates and is, therefore, a promising stationary phase option for this problem. Acetonitrile was found to negatively affect the separation of sugar phosphates on PGC. As it was not required to achieve complete elution of the metabolites tested in this study, acetonitrile was eliminated from the mobile phase, leaving formic acid as the only mobile phase additive in gradient elution. The resulting method achieves baseline separation for 2- and 3-phosphoglyceric acid (*R*_S_ = 1.7) and the pentose phosphates ribose- and ribulose-5-phosphate (*R*_S_ = 2.2). The hexose phosphates fructose-, glucose- and mannose 6-phosphate are not fully baseline-separated (*R*_S_ = 0.5 and 0.9, respectively).

### On-line coupled RP-PGC liquid chromatography

In order to fully exploit the benefits of both RP and PGC selectivities, the low-retained fraction of the RP separation was directly transferred to the PGC column in a heart-cutting on-line two-dimensional setup (Fig. 1). The mobile phase compatibility and fast re-equilibration time of the two stationary phases enabled the use of short gradient programs and a relatively simple instrumental setup. In position A, the flow from the first pump is used to separate sample compounds injected onto the RP column. The PGC column is connected in-line with this flow path after the switching valve, allowing for a direct transfer of the first fraction eluting from the RP column onto the PGC column. After the appropriate fraction transfer time, the valve is switched and the flow from the second pump is now directed to the PGC column in order to elute the transferred analytes. Meanwhile, the flow from the first pump continues to elute components from the RP column. In this way, the RP and PGC separations are performed simultaneously and the effluent streams are combined immediately before introduction into the ESI source. Robust and favorable ionization conditions are ensured *via* the mixing of the two effluent streams. As the maximum system pressure – reached during transfer time – is appr. 320 bar, the use of UHPLC equipment is not required. Overall, this approach is readily implemented requiring only one additional two-position six-port switching valve and an additional HPLC pump.

**Fig. 2:**
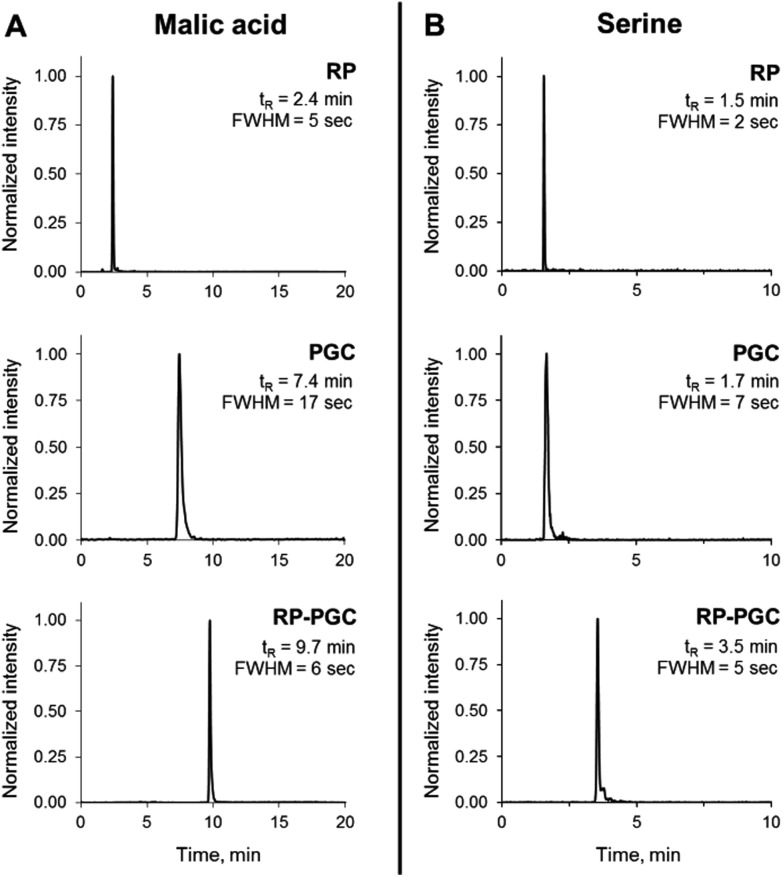
Assessment of peak widths using RP and PGC separately or coupled (RP-PGC), shown exemplarily for malic acid (A) and serine (B). Peak widths are given as full peak width at half-maximal peak height (FWHM). The RP chromatogram shows the peak that is loaded onto the PGC column, while the comparison between PGC and RP-PGC peak widths shows that modulation does not introduce peak broadening.

A mix sample containing 82 relevant intracellular metabolites from the central carbon, energy and redox metabolism (Table 1) was used to assess the method's separation capabilities and metabolome coverage on the level of compound classes as well as individual metabolites. A fully ^13^C-labeled cell extract was added to each standard sample as matrix mimic and for internal standardization purposes. In order to evaluate the gain in metabolite coverage using the presented approach, all samples were analyzed both with RP-PGC-TOFMS and RP-TOFMS. As expected, the combination of RP and PGC liquid chromatography significantly extended the coverage, *i.e.* a significantly higher number of metabolites were retained on either of the chromatographic phases within the same total analysis time of 20 min. Of the 82 metabolites contained in the test mixture, 15 compounds were excluded from further analysis for various reasons, including low electrospray ionization efficiency, poor peak shape and stability issues (especially thiolic metabolites). A total of 30 metabolites eluted from the RP column within less than 2.5 min, among them several amino acids, sugar phosphates and sugar-related compounds. Using the presented RP-PGC method, these metabolites could now be retained and separated on the PGC column and thus be reliably analyzed, while the additional 37 metabolites with RP retention beyond 2.5 min from the original RP separation could be determined simultaneously (Table 1). Band broadening introduced by the suggested instrumental setup was not observed. Instead, narrower peak widths were observed for all peaks analyzed in two dimensions as compared to single-column PGC separation (Fig. 2), presumably because the first dimension acts as a clean-up step to remove the hydrophobic fraction of the sample. The presented RP-PGC method was found to be highly robust and stable, *i.e.* even when the matrix content was increased stepwise to an 8-fold amount, the retention times were still stable within on average 0.4% (short-term precision, *n* = 5 injections, max. 1.1%, Table 1).

**Table 1:** List of 82 metabolites used to assess the metabolome coverage of RP-PGC-TOFMS. 37 metabolites were retained on the RP column and 30 eluted near the void volume of the RP column and were hence automatically analyzed on the second-dimension PGC column. 15 metabolites were excluded from further analysis (“n.a.”). The repeatability precision of retention times was calculated from the observed retention times in repeated injections with a step-wise increase of the matrix load to an 8-fold amount. A cell extract sample from *P. pastoris* was analyzed in an inter-platform comparison of metabolite quantitation employing RP-PGC-TOFMS, RPLC-MS/MS and GC-MS/MS

								*P. pastoris* cell extract, extracted amount, nmol mg_CDW_^–1^					
Abbr.	Metabolite	Polarity	Exact mass, *m*/*z*	RP	PGC	Retention time, min	Retention time RSD (*n* = 5)	RP-PGC-TOFMS		RPLC-MS/MS		GC-MS/MS	
								Average	SD	Average	SD	Average	SD
AAA	α-Aminoadipic acid	+	162.0761		X	5.0	0.1%	1.5	<0.1			1.5	<0.1
Aco	*cis*-Aconitate	+	172.0002	n.a.	n.a.	n.a.	n.a.						
Ade	Adenine	+	136.0618	X		2.7	0.4%	<LOD					
ADP	Adenosine diphosphate	–	426.0221	X		2.7	0.1%	6.9	1.6				
AKG	α-Ketoglutarate	–	145.0142	X		3.0	1.0%	2.8	0.3				
Ala	Alanine	+	90.0550		X	4.2	0.3%	2.2^*a*^	0.1			53	1
3AMP	3′-Adenosine monophosphate	–	346.0558	X		4.7	0.2%	<LOD		<LOD			
5AMP	5′-Adenosine monophosphate	–	346.0558	X		3.1	0.1%	1.4	0.1	2.9	0.1		
Arg	Arginine	+	175.1190		X	3.2	0.7%	58	1				
Asin	Adenosine	+	268.1040	X		5.6	0.3%	<LOD					
Asn	Asparagine	+	133.0608		X	3.7	0.5%	5.3	0.4			5.2	0.1
Asp	Aspartate	+	134.0448		X	4.2	0.4%	12	<1			8.5	0.5
ATP	ATP	+	508.0030	n.a.	n.a.	n.a.	n.a.						
Cit	Citrate	–	191.0197	X		3.6	0.4%	10	<1	9.8	0.1		
5CMP	5′-Cytidine monophosphate	–	322.0446	n.a.	n.a.	n.a.	n.a.						
Cys	Cysteine	+	122.0270	n.a.	n.a.	n.a.	n.a.						
Cys-Gly	Cysteinylglycine	+	179.0485	n.a.	n.a.	n.a.	n.a.						
Cysta	Cystathionine	+	223.0747		X	3.8	0.4%	2.6	0.1			<LOD	
Cyt	Cytosine	+	112.0505	n.a.	n.a.	n.a.	n.a.						
DHAP	Dihydroxyacetonephosphate	–	168.9907	n.a.	n.a.	n.a.	n.a.						
DHIV	Dihydroxyisovalerate	–	133.0506	n.a.	n.a.	n.a.	n.a.						
E4P	Erythrose 4-phosphate	–	199.0013		X	11.9	0.7%	1.7	0.1				
F6P	Fructose 6-phosphate	–	259.0224		X	11.6	0.2%	1.6	0.1			1.3	<0.1
FAD	Flavinadenine dinucleotide	+	786.1644	X		7.1	0.0%	0.1	<0.1				
Fum	Fumarate	–	115.0037	X		9.7	0.4%	2.4	0.1	4.0	0.1	3.4	0.1
G6P	Glucose 6-phosphate	–	259.0224		X	11.8	0.3%	7.2	0.2			6.6	<0.1
GDP	Guanosine diphosphate	–	442.0171	X		9.3	1.1%	<LOD					
Glc-On	Gluconate	–	195.0510		X	6.0	0.7%	0.016	0.001				
Gln	Glutamine	+	147.0764		X	3.9	0.3%	94	1			88	<1
Glu	Glutamate	+	148.0604		X^*b*^	4.3	0.9%						
Glu-Cys	Glutamylcysteine	+	251.0696	n.a.	n.a.	n.a.	n.a.						
Gly	Glycine	+	76.0393	n.a.	n.a.	n.a.	n.a.						
GMP	5′-Guanosine monophosphate	–	362.0507	X		3.6	0.4%	0.2	<0.1	0.21	<0.01		
Gnin	Guanine	+	152.0567	X		2.9	0.4%	<LOD					
GSH	Glutathione, reduced	+	308.0911	X		3.2	0.2%	8.0^*c*^	0.4				
Gsin	Guanosine	+	284.0989	X		5.6	0.5%	<LOD					
GSSG	Glutathione, oxidized	+	613.1592	X		5.3	0.4%	8.1^*c*^	0.2				
Hcys	Homocysteine	+	136.0427	n.a.	n.a.	n.a.	n.a.						
His	Histidine	+	156.0768		X	3.2	1.1%	5.4	0.2				
H-Ser	Homoserine	+	120.0655		X^*d*^	3.7	0.6%						
I-Cit	Isocitrate	–	191.0197	X		2.6	0.4%	0.10	0.02	0.13	<0.01	0.08	<0.01
Ile	Isoleucine	+	132.1019	X		4.4	0.3%	0.036	0.010	0.49	0.01		
Kile	Ketoisoleucine	–	129.0557	X		8.3	0.2%	0.038	0.009				
K-Val	Ketoisovalerate	+	117.0546	X		6.6	0.6%	<LOD				<LOD	
Lac	Lactate	–	89.0244	n.a.	n.a.	n.a.	n.a.						
Leu	Leucine	+	132.1019	X		4.8	0.3%	0.39	0.06	0.39	0.02	0.29	0.03
Lys	Lysine	+	147.1128		X	2.7	0.6%	8.2	0.7			9.5	0.5
M6P	Mannose 6-phosphate	–	259.0224		X	12.4	0.2%	3.4	0.1			2.9	<0.1
Mali	Malate	–	133.0142		X	9.7	0.3%	18	<1	16	<1	17	<1
Man-Ol	Mannitol	+	183.0863		X	4.4	0.6%	0.67	0.15				
Met	Methionine	+	150.0583	X		3.0	0.3%	0.38	0.10	0.48	0.04		
Mt1P	Mannitol 1-phosphate	–	261.0381		X	11.0	0.2%	<LOD				<LOD	
NAD^+^	Nicotinamide adenine dinucleotide, oxidized	+	664.1164	X		4.1	0.4%	2.9^*c*^	0.1				
NADH	Nicotinamide adenine dinucleotide, reduced	–	664.1175	X		5.6	0.7%	9.2^*c*^	0.2				
NADP^+^	Nicotinamide adenine dinucleotide phosphate, oxidized	+	744.0827	X		3.3	0.4%	0.41^*c*^	0.03				
NADPH	Nicotinamide adenine dinucleotide phosphate, reduced	–	744.0838	X		5.6	0.5%	^*c*^					
Oac	Oxaloacetic acid	–	130.9986	n.a.	n.a.	n.a.	n.a.						
OAS	*ortho*-Acetyl serine	+	148.0604		X^*b*^	4.3	0.9%						
PEP	Phosphoenolpyruvate	–	165.9673	n.a.	n.a.	n.a.	n.a.						
2PG	2-Phosphoglycerate	–	184.9857		X	11.5	0.3%	0.083	0.005			<LOD	
3PG	3-Phosphoglycerate	–	184.9857		X	11.9	0.2%	0.75	0.03			0.51	0.03
6PGA	6-Phosphogluconate	–	275.0174		X	14.0	0.1%	0.41	0.01			0.28	0.05
Phe	Phenylalanine	+	166.0863	X		6.1	0.8%	0.34	0.01	0.40	0.01	0.42	0.02
Pro	Proline	+	116.0706		X	4.1	0.2%	<LOD				11	<1
Pyr	Pyruvate	–	87.0088	X		2.6	0.3%	0.97	0.06				
R5P	Ribose 5-phosphate	–	229.0119		X	11.2	0.5%	0.66	0.03			1.8	<0.1
Ri-Fl	Riboflavin	+	377.1456	X		7.6	0.1%	<LOD		<LOD			
Rl5P	Ribulose 5-phosphate	–	229.0119		X	11.7	0.2%	0.34	0.04				
S7P	Sedoheptulose 7-phosphate	–	289.0330		X	12.2	0.3%	2.6	0.2			2.7	0.1
SAH	*S*-Adenosylhomocysteine	+	385.1289	X		5.5	0.4%	0.089	0.019				
SAM	*S*-Adenosylmethionine	+	399.1451	X		4.2	0.8%	<LOD					
Ser	Serine	+	106.0499		X	3.5	0.6%	4.8	1.9			4.2	0.2
Suc	Succinate	–	117.0193	X		4.6	0.6%	1.9	0.1	2.0	0.1	1.6	0.1
Thi	Thiamine	+	265.1118	n.a.	n.a.	n.a.	n.a.						
Thr	Threonine	+	120.0655		X^*d*^	3.7	0.6%						
Thy	Thymine	+	127.0502	X		5.7	0.7%	<LOD					
Trp	Tryptophan	+	205.0972	X		6.7	0.2%	0.093	0.006	0.077	0.001		
Tyr	Tyrosine	+	182.0812	X		4.6	0.4%	0.41	0.03	0.53	<0.01	0.46	0.07
5UMP	5′-Uridine monophosphate	–	323.0286	X		3.0	0.3%	0.54	0.11	0.26	0.03		
Ura	Uracil	+	113.0346	X		3.1	0.4%	<LOD					
Uri	Uridine	+	245.0768	X		5.0	0.1%	<LOD					
Val	Valine	+	118.0863		X	4.7	0.6%	2.6	0.2	2.2	0.1		

^*a*^Poor linear calibration due to strong interaction with stationary phase at low concentrations.

^*b*^Glutamic acid and *ortho*-acetyl serine co-elute.

^*c*^The reliable determination of intracellular NAD^+^, NADH, NADP^+^, NADPH, GSH and GSSG levels requires dedicated sample preparation methods.^45,46^ The validated RPLC-MS/MS methodology including sample preparation does not include these metabolites, therefore reference values are not available.

^*d*^Homoserine and threonine co-elute.

The above-mentioned separation of sugar phosphates and related metabolites was also accomplished in this setup, extending the metabolome coverage of the overall method by a full compound class of high biological significance. Moreover, a wide range of amino acids and their derivatives, nucleobases, nucleosides and nucleotides, vitamins and cofactors are also captured with this approach (Table 1). Except for homoserine and threonine as well as glutamic acid and *o*-acetyl serine, all isobaric overlaps could be resolved chromatographically. Nevertheless, the scope of this method is clearly not limited to the metabolites tested with chemical standards in this study. As it provides retention for both hydrophobic and polar compounds, this analytical platform can be expected to cover many more intracellular metabolites from various compound classes, hence the method is also highly suitable for metabolic fingerprinting.

The extension of the separating power using PGC according to the described approach has some distinct advantages over other possible options such as ion exchange chromatography (IC) or ion pair chromatography. IC methodologies typically require a de-salting device for compatibility with MS and suffer from limited metabolome coverage and sensitivity. While ion pair chromatography can offer excellent separations of polar metabolites, its application also requires a dedicated mass spectrometer for hyphenation due to the in most cases irreversible contamination of both HPLC systems and MS ion optics.

### RP-PGC-MS in metabolic profiling

The accurate quantification of intracellular metabolites is an important tool in metabolomics, albeit fraught with complications. As isotope dilution-type techniques for internal standardization and intensity correction became state-of-the art in metabolic profiling, many of these problems were solved. Nonetheless, accurate quantitation in complex biological matrices relies on robust and efficient chromatographic separation methods in order to ensure sufficient data quality. All cell extracts analyzed with RP-PGC-TOFMS in this study were also analyzed using both RPLC-MS/MS and GC-MS/MS. Wherever possible the quantitative results obtained from the different platforms were compared (Table 1). In fact, the obtained results were comparable and in satisfactory agreement, considering the typical total combined measurement uncertainty associated with such analysis.^42,47^ The standard deviations given in Table 1 for RP-PGC-TOFMS were determined from the observed relative standard deviations in repetitive injections (*n* = 5) of a quality control sample across a time span of 20 h and demonstrate the method's good repeatability. As can be readily observed only a small number of metabolites needs further elucidation, such as *e.g.* alanine showing strong interactions with the stationary phase on PGC, which resulted in a poor linear calibration. Remarkably, the high degree of inter-platform agreement was accomplished despite the fact that TOFMS is not the ideal platform for quantitation tasks, as both the selectivity in the *m*/*z* dimension and the linear dynamic range are typically limited with respect to triple quadrupole MS. Hence, the overall performance of the presented RP-PGC separation approach can be regarded as excellent.

## Conclusion

Liquid chromatography using porous graphitized carbon as stationary phase is a promising approach for metabolomics applications. It provides good retention and a distinct selectivity for compounds that cannot be retained or separated by conventional RP or HILIC. Chromatography on PGC hence complements the analytical toolset typically employed in metabolomics. The presented combined RP-PGC method provides broad coverage across different compound classes within the metabolome, as was demonstrated exemplarily on a set of 82 test metabolites from central carbon, energy and redox metabolism. Most importantly, this separation system allows for the recovery of directly biologically relevant information that is lost in conventional analytical workflows as weakly retained compounds are typically excluded from data evaluation. Comparable coverage across as many metabolites of different polarity in LC has only been achieved in literature using ion pair LC, which is subject to severe shortcomings in its practical application. The presented heart-cut two-dimensional setup requires only one additional two-position six-port valve for modulation and an additional HPLC pump, hence the instrumental setup is flexible and straightforward in its implementation.

The prominent tasks in metabolomics are metabolic profiling, *i.e.* the quantitative assessment of the intracellular metabolome, and metabolic fingerprinting, *i.e.* the generation of representative snapshots of cellular metabolism in a certain physiological state. Even though the analytical mindset is different in these two approaches, the presented method meets the requirements for both, in that it is stable, robust towards the presence of matrix and highly reproducible. A platform inter-comparison with GC- and LC-tandem MS also demonstrated the method's suitability for both absolute and relative quantitation tasks. This work moreover exemplifies that in times of very high resolution mass spectrometry, chromatographic selectivity is still a key aspect in the development of successful analytical platforms for complex sample types such as cell extracts.
